# Book Reviews

**Published:** 1895-04

**Authors:** 


### Book Reviews.

Bureau of Education. Contributions to American Educational History. Edited by Herbert B. Adams. No. 14, the History of Education in Connecticut by Bernard C. Steiner, A. M. (Yale), Instructor in History and Ph. D., Johns Hopkins University, Librarian of the Enoch Pratt Free Library of Baltimore City. Washington: Government Printing Office. 1893.

Report No. 14, prepared by the National Bureau of Education, is a review of the history of the higher institutions of learning in Connecticut. It is largely occupied with a narrative of the beginnings in colonial days, and the later development of Yale university. Connecticut puritanism might have been narrow on some sides of its social and religious life, but its devotion to higher learning, its sacrifices to found and maintain educational institutions have given to the state a wholesome broadness, and have placed its leading university in the foremost rank of the great universities of the world.

The original charter of Yale college was granted A. D. 1701, naming ten congregational clergymen trustees, of whom all but one were Harvard graduates. The location was left open, but New Haven finally won in 1718. Yale university was born in an intensely theologic age, and amid struggles of the colonies with poverty, and when the French and Indian wars were a serious embarrassment. Several men of letters in England interested themselves in the enterprise. Sir Richard Steele, Isaac Newton, Bishop Berkeley and others were contributors to its library. The college derived its name by a happy thought of one of its trustees.

Elihu Yale, the son of a Boston merchant, sought his fortune in India. He became governor of Madras and accumulated large wealth. He is said to have been "unscrupulous in conduct and of lax morality," but with redeeming qualities. It was suggested to him by Cotton Mather that he might give his name to the new college. The gift of £800 sterling was the response, and the Madras governor won an easy immortality. No graduate of Yale, of even twenty-five years ago, remembering its four plain dormitories and recitation rooms, notes its new and magnificent edifices for each of its departments, its army of students and professors, its advance in every department of literature and science, but feels an emotion of pride as he realizes the greatness and the renown of his Alma Mater.

The college had early in its history theologic controversies which threatened for a time its congregational control. The honorable part of the college during the period of the war of the revolution, and of our civil war through its students and officers, and the great part it has had in every department of professional, public and civic life, is dwelt upon at length in the report as well as its ever-widening development as a university.

A very interesting account follows of the founding of Trinity college, at Hartford, in 1824, under the auspices of the Episcopal church. As early as 1788, preliminary steps were taken to establish a college for the furtherance of the educational and religious interests of that church.

This is followed by an equally interesting sketch of the Wesleyan university, established in Middletown in 1831.

The Hartford Theological college grew out of the theological controversy in the '30's and '40's, between Professor Taylor, of the Yale divinity school, and Rev. Doctor Tyler, of East Windsor. This battle royal over theologic metaphysics, which, at the time, divided the Congregational church of Connecticut into two hostile camps, has passed into history, leaving neither victors nor vanquished. Honest and earnest as were these great polemics, they spent their forces over questions now superseded by the much larger interests and more practical questions which occupy the leaders and people of Christian churches.

The report closes with a notice of Berkley Divinity school in Middletown, founded in 1868. This admirable report presents

Connecticut in the forefront of the educational history of the country.

---

DISEASES OF THE EAR. A Text-Book for Practitioners and Students of Medicine. By EDWARD BRADFORD DENCH, Ph. B., M. D., Professor of Diseases of the Ear in the Bellevue Hospital Medical College; Aural Surgeon, New York Eye and Ear Infirmary, etc., etc. Octavo, pp. xxii.—645. With eight colored plates and 152 illustrations in the text. Price, cloth, $5.00; leather, $6.00. New York: D. Appleton & Co. 1894.

The numerous works on the ear which have recently been published would convey the impression that there could be no demand for another new treatise. But it is only necessary to read the work of Dr. Dench to be convinced that it fulfils a purpose more completely than many others in presenting the latest information and the most advanced therapeutics in otology.

The work is divided into five sections, as follows:

I. The anatomy and physiology of the ear.

II. Diseases of the conducting apparatus.

III. Surgery of the conducting apparatus.

IV. Diseases of the perceptive mechanism.

V. Complicating aural affections.

In section I., a very excellent description is given of the anatomy and physiology, including the physical and functional examination of the ear. Especial emphasis is laid upon the importance of functional examination and the results of the most recent investigations are placed at the disposal of the reader in such a manner as to enable him to use them in diagnosis.

Section II. treats of diseases of the auricle, external auditory canal, diseases of the middle ear and diseases of the mastoid process. This section is the largest of the work and includes subjects of the greatest practical interest to the practitioner, among which we may mention catarrhal and purulent inflammations of the middle ear, mastoiditis and intra-cranial complications of tympanic inflammations. The author has availed himself here of the most reliable resources in diagnosis and lays down most trustworthy principles as to prognosis and treatment.

In the next section (Section III.) he considers surgical operations on the middle ear, mastoid process, and brain, and gives detailed and clear descriptions of myringotomy, myringectomy, division of the tensor tympani, anterior ligament of the malleus, intra-tympanic adhesions, excision of the ossicles, stapedectomy, Stacke's method of exposing the tympanic cavity and mastoid antrum, the later methods of opening the mastoid process, and the procedures by which to expose the lateral sinus, the brain in cerebral abscess, and the like. There is a fund of information, the most practical and recent, gathered into this section, of extreme value and which we believe is nowhere else to be found within the same space in the English language.

Section IV. contains an excellent presentation of internal-ear diseases and embodies many new facts that have not heretofore been collected.

Section V. reviews complicating aural affections and includes aural affections complicating the acute infectious diseases; those dependent upon chronic visceral conditions, such as nephritis, tuberculosis, leucemia, diabetes, gout, rheumatism, medicinal substances; disturbances dependent upon functional nervous diseases, such as neurasthenia and hysteria; reflex disturbances; deaf-mutism. It concludes with several chapters on diseases of the nose and naso-pharynx, briefly but very practically considered in their relation to affections of the ear.

This brief summary will give the reader but an insufficient idea of the scope and character of this work. It is both comprehensive and scholarly and represents the latest in otological thought and practice. It is well illustrated and is written in a most charming style. A. A. H.

---

History of the Life of D. Hayes Agnew, M. D., LL. D. By J. Howe Adams, M. D. With fourteen full-page portraits and other illustrations. In one large royal octavo volume, 376 pages, extra cloth, beveled edges, $2.50 net; half-morocco, gilt top, $3.50 net. Sold only by subscription. Philadelphia: The F. A. Davis Co., Publishers, 1914 and 1916 Cherry street.

Few men, in their day, have had more reverent and loving friends than Dr. Agnew. And any story of his life and work is always read with the keenest interest, for in the record one was certain to find the impress of a truly great character. Some of the simplest incidents in his life were most illustrative of the man. So this work of Dr. Adams will find many appreciative readers, who cannot fail to know Dr. Agnew the better for having read it. The book is written according to the author's conception of what Dr. Agnew would have dictated. He expresses his idea of what a biographer should be and do in the following words:

> A biographer, in preparing the life of his hero, loves to dwell chiefly on those efforts and achievements which have attracted public admiration—on those master-strokes in the life of his subject which have raised him above the level of his fellow-men—leaving out those lesser characteristics which not only serve to consolidate the character and lead to success, but which, from their multiform points in which they come in contact with common humanity, confer a personal interest in the life.

In this spirit the writer has told us of the ancestry of Dr. Agnew, of his early struggles in medicine and business, of his career as a practitioner, teacher and author, of home life and final days. While the method employed by Dr. Adams has many commendable features and distinct advantages in giving us an intimate knowledge of the character delineated, it has also the danger of prolixity and of injudicious minuteness of detail, and this danger, it must be confessed, Dr. Adams has not always foreseen and avoided. To say, for instance, that Dr. Agnew smoked the very best brand of cigars and cigarettes, to tell what he ate for breakfast, to state that his ice house was filled each winter with a particularly pure ice sent by a patient, and the like, seems to detract from the dignity of the record of a life so full of great thoughts and deeds. Notwithstanding such blemishes the book is a good one, because written by one who *knew* his hero. In this story, simply told as it is, there is much that is new, but always characteristic of that remarkable man to whom the medical profession will ever owe a debt of gratitude. J. P.

---

THE JOHNS HOPKINS HOSPITAL REPORTS. Volume IV., Nos. 4 and 5. Report in Neurology II. Baltimore: The Johns Hopkins Press. 1894.

This report is devoted entirely to careful scientific investigations of Professor Henry J. Berkley in the field of neurology, and shows an immense amount of research and scientific thought. It is, perhaps, as good an index as can be given of the excellent original work done in the Johns Hopkins laboratories, and does not suffer in comparison with that done in the best European laboratories.

The first article is devoted to the study of dementia paralytica in the negro race, with report of five cases coming under the personal observation of the author. These cases were carefully examined on the post-mortem table, and in two of them a most careful microscopical examination was made of the tissues of the brain and cord.

The remarkably low percentage of paresis among the colored people is dwelt upon, and another important fact is the absence of syphilis in these cases, also the absence of alcoholism. The author rather believes that the cause of paresis is due to the altered condition of the race from a life of comparative freedom to the one for the struggle of individual existence.

The most characteristic symptom for the disease in the negro is the extreme rapidity of mental enfeeblement. Pathologically, the author believes that the beginning of the disease is to be found in some alteration of the blood supply, followed by a peri-arterial lymphoid growth, disturbance of the lymph currents, malnutrition, degenerative changes, atrophy of the nerve elements and overgrowth of the spider cells. The author does not believe that paresis is due to a diffuse interstitial cortical encephalitis, in which the connective tissue elements are primarily affected, nor that it is a diffuse parenchymatous inflammation which commences in the nerve elements and involves, secondarily, the neuroglia structures.

The succeeding articles are devoted to a study of the intrinsic nerves of the liver, including the gall capillaries of the rabbit's liver; the privascular cells of the rabbit's liver; the intrinsic pulmonary nerves in mammalia; the intrinsic nerve supply of the cardiac ventricles in certain vertebrates; the intrinsic nerves of the sub-maxillary gland of the rat; the intrinsic nerves of the thyroid gland of the dog; and the nerve elements of the pituitary gland. These papers are all accompanied by beautiful plates, showing the course and distribution of the nerve fibrils and their termination in the special tissues. A review of these labors would necessarily be too exhaustive for the JOURNAL, and the reader interested in these subjects must be referred to the original reports.

W. C. K.

---

PRACTICAL URINALYSIS AND URINARY DIAGNOSIS: A Manual for the Use of Physicians, Surgeons and Students. By CHARLES W. PURDY, M. D., Queen's University; Fellow of the Royal College of Physicians and Surgeons, Kingston; Professor of Urinology and Urinary Diagnosis at the Chicago Post-Graduate Medical School; Author of Bright's Disease and Allied Affections of the Kidneys; also of Diabetes: Its Causes, Symptoms and Treatment. With numerous illustrations, including photo-engravings and colored plates. In one crown 8vo volume, 360 pages, in extra cloth, $2.50 net. Philadelphia: The F. A. Davis Co., 1914 and 1916 Cherry street. 1894.

The importance of a knowledge of the subjects treated of in this book cannot be over-estimated. The subtle beginning of most diseases of the kidneys is a serious bar to their intelligent early treatment. The kidney deserves to be more universally, and, we might add, more thoroughly studied. It is a great eliminator of the ashes of combustion, and when its offices are imperfectly performed, the economy suffers in many and divers ways.

The analysis of urine is considered in Part I. of this work, which comprises the first 228 pages. Physiological chemistry, as applied to this subject, is dealt with in much detail. This is especially true with reference to the sections devoted to the consideration of the abnormal constituents of the urine.

Part II. is devoted to urinary diagnosis, with which subject every physician should become familiar. An appendix is added to the book, which treats of the examination of urine for life insurance. Every examiner should familiarize himself with this important branch of the subject Life insurance companies might, with great propriety, reprint this section and distribute it among their examiners. A good index closes this well-printed, interesting and valuable treatise, which we commend as one of the best of its kind.

---

FOURTEENTH ANNUAL REPORT OF THE STATE BOARD OF HEALTH OF NEW YORK. In two volumes and accompanying maps. Transmitted to the Legislature February 15, 1894. Albany: James B. Lyon, State Printer. 1894.

These volumes constitute a valuable record of the official transactions of the state board of health, its subordinates and auxiliaries for the year 1893. Probably one of the most interesting subjects dealt with is that of tuberculosis in cattle. The growing importance of this question and the bearing it has upon the health of the community renders it essential that the state board of health shall be clothed with ample power to deal summarily with tuberculous animals, singly or in herds, without recourse. All such animals should be slaughtered and cremated at the expense of the owners. This is the least possible punishment that the state can inflict upon an owner of such dangerously diseased cattle. It might be as well understood first as last that the propagation of consumption through animal food is a serious matter, and whoever becomes a party to this method of spreading infectious disease must be made to suffer, at least, the condemnation and destruction of the diseased animals. The second volume of this report is devoted to this subject and experiments with tuberculin are carefully recorded.

The board of health appears to be pursuing its investigations with caution on scientific lines.

---

Differential Diagnosis and Treatment of Diseases of the Eye. By A. E. Adams, M. D., Instructor in Diseases of the Eye in the Post-Graduate Medical College; Assistant Surgeon to Manhattan Eye and Ear Hospital, New York; Ophthalmic Surgeon to St. Luke's Hospital, and Consulting Ophthalmologist to the Home of the Friendless, Newburgh; Fellow of the New York Academy of Medicine, etc., etc. Duodecimo, pp. 94. Price, $1.00. New York: G. P. Putnam's Sons. 1894.

This little work is made up of tables of symptoms and indications of treatment of diseases of the eye. "They are designed more especially for the active practitioner who does not claim to be an ophthalmologist or even well posted on diseases of the eye" (Preface).

We fear that, instead of serving the purpose intended, these tables will only lead to confusion and uncertainty in diagnosis and treatment. A. A. H.

---

Text-Book of Medical and Pharmaceutical Chemistry. By Elias H. Bartley, B. L., M. D., Professor of Chemistry and Toxicology in Long Island College Hospital; Dean and Professor of Organic Chemistry in the Brooklyn College of Pharmacy, etc., etc. Third edition, revised and enlarged with eighty-four illustrations. Small 8vo, pp. xiv.—684. Price, $3.00. Philadelphia: P. Blakiston, Son & Co., 1012 Walnut street. 1894.

This edition is considerably larger than the second edition, which was reviewed in the Journal some time since.

The description of a large number of synthetical compounds, some new tables of analytical tests, and a chapter on physiological and clinical chemistry have very materially improved the work.

The typography, plates and general make-up of this edition is excellent. J. A. M.

ATTFIELD'S CHEMISTRY. Fourteenth edition. Chemistry: General, Medical and Pharmaceutical; including the Chemistry of the U. S. Pharmacopeia. A Manual of the General Principles of the Science and their Application to Medicine and Pharmacy. By JOHN ATTFIELD, M. A., Ph. D., F. I. C., F. C. S., F. R. S., etc., Professor of Practical Chemistry to the Pharmaceutical Society of Great Britain, etc. Fourteenth edition, specially revised by the author for America to accord with the new U. S. Pharmacopeia. In one handsome royal 12mo volume of 794 pages, with eighty-eight illustrations. Cloth, $2.75; leather, $3.25. Philadelphia: Lea Brothers & Co. 1894.

Attfield's Chemistry has been too long before the profession to need a special introduction or an extended resumé of its merits.

This edition, the fourteenth, is revised in accordance with the scheme of the new United States Pharmacopeia, and is printed concurrently with the fifteenth British edition.

The revision is thorough; new processes are added, faulty and obsolete ones are dropped. The same thoroughness which characterized the previous editions is amplified in the present one.

It is safe to say that the new edition will establish Attfield's Chemistry in the high place it occupies in the estimation of the profession, more firmly than ever. J. A. M.

---

SYLLABUS OF GYNECOLOGY, based on the American Text-Book of Gynecology. By J. W. LONG, M. D., Richmond, Professor of Gynecology and Pediatrics in the Medical College of Virginia; Gynecologist to the Hospital of the Medical College of Virginia; Consulting Gynecologist to the Richmond City Dispensary, etc., etc. Price, $1.00 net. Philadelphia: W. B. Saunders, 925 Walnut street. 1895.

The author asserts that he has written this syllabus with a three-fold object: first, to be used as lecture notes; second, to enable the student more intelligently to follow and remember lectures; and, third, as a convenient reference for practitioners of medicine. The book is to be commended from the three points of view named. Dr. Long is an able teacher, an acute observer and a logical reasoner. He has produced in this work the most comprehensive syllabus of gynecology yet brought to our notice. We observe that he makes use of the word celiotomy, though, to his credit, in a limited degree. We have expressed our opinion on this subject heretofore, hence need not repeat our criticism of the term at this time. It is such bad form, however, that we cannot refrain from an allusion to its use in so good a book as this. We hope to see the term expunged in future editions.

---

### BOOKS RECEIVED.

Manual of Chemistry. A Guide to Lectures and Laboratory work for Beginners in Chemistry. A Text-book specially adapted for Students of Pharmacy and Medicine. By W. W. Simon, Ph. D., M. D., Professor of Chemistry and Toxicology, College of Physicians and Surgeons, Baltimore; Professor of Chemistry in the Maryland College of Pharmacy. New (fifth) edition. In one octavo volume of 502 pages, with forty-four engravings and eight colored plates illustrating sixty-four of the most important chemical tests. Cloth, $3.25. Philadelphia: Lea Brothers & Co. 1895.

Transactions of the Medical Society of the State of North Carolina. Forty-first annual meeting, held at Greensboro, N. C., May 15, 16, 17, 1894. Wilmington, N. C.: Jackson & Bell. 1894.

A Manual of Bandaging. Adapted for Self-Instruction. By C. Henri Leonard, A. M., M. D., Professor of the Medical and Surgical Diseases of Women, and Clinical Gynecology in the Detroit College of Medicine. Sixth edition, with 139 engravings. Cloth, 8vo, 189 pages. Price, $1.50. Detroit, Mich.: The Illustrated Medical Journal Co., Publishers.

Suggestions to Hospital and Asylum Visitors. By John S. Billings, M. D., Director of the Hospital of the University of Pennsylvania, and Henry M. Hurd, M. D., Superintendent of the Johns Hopkins Hospital. With an introduction by S. Weir Mitchell, M. D. Price, 50 cents. Philadelphia: J. B. Lippincott Co. 1895.

The Physicians' Vade Mecum. Being a Hand-book of Medical and Surgical Reference, with other Useful Information and Tables. By Sebastian J. Wimmer, M. A., M. D., Author of Tables and Notes on Human Osteology, etc. With additions by Frank S. Parsons, M. D. Price, $1.00. Philadelphia: The Medical Publishing Co. 1894.

Suggestive Therapeutics in Psychopathia Sexualis, with Especial Reference to Contrary Sexual Instinct. By Dr. A. von Schrenck-Notzing (Munich, Germany). Authorized translation from the German, by Charles Gilbert Chaddock, M. D., Professor of Diseases of the Nervous System, Marion-Sims College of Medicine, St. Louis; Member of the American Medico-Psychological Association; Attending Neurologist to the Rebekah Hospital, St. Louis, Mo., etc., etc. One volume royal 8vo, 325 pages. Extra cloth, $2.50 net; sheep, $3.50 net. Sold only by subscription to the medical profession exclusively. Philadelphia: The F. A. Davis Co., Publishers, 1914 and 1916 Cherry street.

---

### Correspondence.

### THE CHARCOT MONUMENT FUND.

BUFFALO, March 25, 1895.

*To the Editor of the Buffalo Medical Journal:*

SIR—The following is a statement of the contributions received to date to the Charcot monument fund:

BUFFALO.

| | |
|---|---|
| Dr. Wm. C. Krauss | $5.00 |
| Dr. Chas. S. Jones | 5.00 |
| Dr. James W. Putnum | 5.00 |

New York City, $290 ; Philadelphia, $150 ; Baltimore, $50 ; Pittsburg, $58 ; Montreal, $19 ; Boston, $35 ; Denver, $10 ; St. Louis, $10 ; Chicago, $5 ; total, $642.

It is requested that persons living in Buffalo and vicinity who wish to add to the fund, send their contributions to the undersigned without delay.

JAMES WRIGHT PUTNAM, M. D.,
388 Franklin street,
*Member of the Committee from Buffalo.*

---

